# Why It Appeals to Me

**Published:** 1920-12-25

**Authors:** 


### "THE HOSPITAL CHRISTMAS."

### Why it Appeals to Me.

No one who has ever spent a Christmas in hospital can help feeling that there is something unique about it; something different from anything else they have experienced; something they will never forget; but something, withal, difficult to describe.

It is with the object of endeavouring to find the secret of this wonderful "something" that the following article has been got together; in which the various views of those connected with institutions of different size and character are expressed in answer to the question "Why does a hospital Christmas appeal to you?"

### What a Matron Says.

"If I am to speak quite candidly—from an individual's point of view I am afraid a hospital Christmas is rather an anxiety with added responsibilities for any matron; at the same time it is always a great pleasure and worth all the trouble to see the happiness of the patients and staff.

"The 'atmosphere' is so wonderful, even the very ill patients seem to feel it and take an interest in the doings, and it seems to brighten their lives for at least a short time, although they may be too ill to take much notice of anything about them on other days. We had a very marked incident of this kind a year or two ago; a patient who was obviously not going to get better, and knew that he was not going to do so, asked that he might get into a wheeled-chair and go round and see everything, as it would be his last Christmas here. He was allowed to do this, and it was remarkable how he entered into everything and seemed to enjoy the day. About a week afterwards he died, but during the whole of that time he could do nothing but talk about the happy time he had had on Christmas Day. Such an incident shows why we are glad that Christmas comes at least once a year, however great and extra the responsibilities it may bring with it for those concerned."

### A Sister's Thoughts.

"Having spent many years in hospital, I can truly say that the recollections of those times include some of my happiest Christmas Days. The season appeals in many ways, amidst the hush of Christmas Eve, when the last strand of holly and evergreen has been hung up, and before Santa Claus comes to visit us with his gifts—when from the dim corridors the strains of the carol singers remind us that "unto us a Son is given"—we can enter into the spirit of a hospital Christmas. There is the peacefulness of the early Eucharist—then the stir and excitement of the day, and if at its close when the lights are low in the ward someone whispers, 'Sister, it's been a splendid day this Christmas in hospital,' one feels that the efforts to brighten lives of pain and cheer sad hearts, if only for one day, have not really been in vain."

### The Visitor's Opinion.

"I think the hospital Christmas appeals to me because the ordinary things seem to be forgotten in the remembrance of the day; there seems no thought of medicines or bandages or splints; operations and anæsthetics and casualty rooms seem to have been put away, while the men and women smile and the children laugh as though their pain and suffering was a thing of the past and out of mind—and it is nice for us visitors to forget sometimes, nice to forget our own little worries, nice to forget the sadness of life, nice to forget its pettiness, nice to forget self!"

### What a Chaplain Feels.

I heard the story told from a pulpit one Hospital Sunday of a little slum lad who had met with an accident and was taken unconscious to one of our large institutions on Christmas Eve. Soon after his admission he regained consciousness, and looking around the wonderfully decorated ward and at the house surgeon, who in white coat was bending over him, said, "Please, Sir, is this heaven, and are you God?" It is something of that spirit that marks a hospital Christmas—a spirit removed far above that usually met with, alas, in daily life! There is no fault finding, no criticising, no thought of self—everyone seems to go out of their way to make everyone else happy; the patients smile even in pain; there is never a grumble but a feeling of harmony and love; there is a real breath of the spirit of goodwill and peace—a seemingly real grasp of the Great Festival's message—Emmanuel.

